# Systems biology approach to stage-wise characterization of epigenetic genes in lung adenocarcinoma

**DOI:** 10.1186/1752-0509-7-141

**Published:** 2013-12-26

**Authors:** Meeta P Pradhan, Akshay Desai, Mathew J Palakal

**Affiliations:** 1 School of Informatics and Computing, Indiana University Purdue University Indianapolis, Indianapolis IN, USA

**Keywords:** Epigenetic genes, Stages, LUAD, TFs, Subnetwork, NSCLC, SCLC

## Abstract

**Background:**

Epigenetics refers to the reversible functional modifications of the genome that do not correlate to changes in the DNA sequence. The aim of this study is to understand DNA methylation patterns across different stages of lung adenocarcinoma (LUAD).

**Results:**

Our study identified 72, 93 and 170 significant DNA methylated genes in Stages I, II and III respectively. A set of common 34 significant DNA methylated genes located in the promoter section of the true CpG islands were found across stages, and these were: *HOX* genes, *FOXG1*, *GRIK3*, *HAND2*, *PRKCB*, etc. Of the total significant DNA methylated genes, 65 correlated with transcription function. The epigenetic analysis identified the following novel genes across all stages: *PTGDR*, *TLX3*, *and POU4F2*. The stage-wise analysis observed the appearance of *NEUROG1* gene in Stage I and its re-appearance in Stage III. The analysis showed similar epigenetic pattern across Stage I and Stage III. Pathway analysis revealed important signaling and metabolic pathways of LUAD to correlate with epigenetics. Epigenetic subnetwork analysis identified a set of seven conserved genes across all stages: *UBC*, *KRAS*, *PIK3CA*, *PIK3R3*, *RAF1*, *BRAF*, *and RAP1A*. A detailed literature analysis elucidated epigenetic genes like *FOXG1*, *HLA-G*, *and NKX6-2* to be known as prognostic targets.

**Conclusion:**

Integrating epigenetic information for genes with expression data can be useful for comprehending in-depth disease mechanism and for the ultimate goal of better target identification.

## Background

Cancer progression is associated with mutation and differential gene expression [1]. Many oncogenes and tumor suppressor genes responsible for cancer are linked to mutations [2]. Besides these mutations, recent studies correlate epigenetic features to play an important role in cancer development and propagation [3-10]. Epigenetics refers to all gene modifications except the change in the DNA sequence [11]. These modifications are caused by changes in the chromatin structure [11], DNA methylation, and histone modifications. Nearly 50% of human genes are associated with CpG islands in the promoter regions [12]. If these promoter regions undergo methylation, they lead to disease progression [12-14]. DNA methylation occurs in and out of CpG islands, which in a normal tissue is entirely unmethylated at all stages of development and allows gene expression if a transcription factor (TF) is present [15]. These changes affect the binding of transcription factors (TFs) to DNA [16]. This occurs by DNA methylation of the 5′-CG-3′ pair usually observed at the transcription regulation sites, which results in silencing or activation of the downstream genes [17].

Advances in next-generation technologies have led to identification of genome-wide DNA methylations in a large number of disease samples. Methylation sites have been analyzed based on clustering with respect to genomic regions, methylation patterns, and common regulatory patterns [16]. Increased methylation of CpG islands in the promoter regions known as hypermethylation leads to silencing of genes, usually associated with tumor suppressor genes [18], whereas the decreased methylation known as hypomethylation is associated with gene overexpression i.e., activation of oncogenes [18]. Both hypermethylation and hypomethylation are known to be linked to tumors, autoimmune and other diseases [16,19,20]. In cancers, many genes are methylated in normally unmethylated promoter CpG islands, eventually influencing transcriptional activity both in early and late stages [19,21,22]. Specific examples of DNA methylation role in cancers include hypermethylation of *BRCA1* in breast and ovarian cancer [22,23]; *DOK7* in breast cancer [22]; *MYOD1* in hematological neoplasm; *APC*, *HOX2*, *OTX1* genes in non-small cell lung carcinoma (NSCLC) [24]; FEN1 in breast tumor cells [25]; and hypomethylation of TKTL1 in head and neck squamous cell carcinoma [26]. Literature provides evidence correlating transcriptional activities with methylated genes [12], suggesting the role of higher methylation in lowering the transcriptional activity [4]. Since these epigenetic alterations are reversible, identification of methylated genes for targeted modifications in cancer can provide a new approach to successful drug therapies.

Lung cancer is one of the most commonly diagnosed cancers in United States. Lung cancer is morphologically divided into NSCLC and small cell lung cancer (SCLC) [27]. NSCLC is classified into three major histopatholgical subtypes: adenocarcinoma, squamous cell carcinoma and large cell carcinoma. Lung adenocarcinoma (LUAD) is currently the most common of the lung cancers in both smokers and non-smokers. LUAD is classified into four stages: Stage I, when the cancer is localized; Stage II, when the cancer has spread to the lymph nodes; Stage III, when the cancer has spread to tissues near the lungs; and, Stage IV, when metastasis has occurred [28]. Few DNA methylation studies have been reported for NSCLC, and the DNA methylated genes identified in these were *APC*, *CDH13*, *CDKN2A*, *DAPK*, *hMLH1*, *HOX*, *OTX1*, *HOX2*, *ZIC4*, and *RASSF1*[24,27,29,30]. There have been no stage-wise methylation studies reported on LUAD. It has been observed that LUAD is highly heterogeneous, and there is less similarity between stages and across the samples within the stages [24], therefore understanding DNA methylated genes profile across LUAD can provide a new insight.

The aim of the study was to elucidate the DNA methylation patterns across different stages of LUAD from publicly available data resources. We used The Cancer Genome Atlas Data (TCGA) as our resource for methylation data. In this study, a systems biology approach of integrating gene-expression, DNA methylation and protein-protein interaction data for finding highly important DNA methylated genes across stages of LUAD were developed. These DNA methylated genes were compared across stages for their uniqueness and commonality to identify the patterns across stages of LUAD. These patterns were then validated and ranked for their importance in LUAD using literature evidences [3,31]. These ranked patterns were analyzed as potential targets of LUAD. The limitation of the study was the laboratory validation of the targets and availability of datasets in the TCGA. To our knowledge this is the first study that explains the DNA methylated genes across stages of LUAD.

## Results

The objective of this study was to understand the Significant DNA methylated genes across the four stages of LUAD and analyze them as potential targets. The TCGA data associated with LUAD was classified based on these stages. For this stage-wise data, the patient’s age ranged from 58–75 with few outliers. The Significant expressed genes and Significant DNA methylated genes were identified based on the *p-values* and beta-values for each stage as described in the methodology. Resampling technique were performed for the correction and these provided the set of *p-values*. Using the technique used in paper [32], *p-value* of 0.0012 was obtained from *q-values*. Using this cutoff the Significant DNA methylated genes were re-evaluated and overlap between the previous and resampled results were calculated. A substantial amount of overlap between old and new set of Significant DNA methylated genes were observed. Additional file 1 shows the *p-value* correction for original and corrected Stage I data after resampling. The Significant DNA methylated genes were then further classified as hypermethylated and hypomethylated (methodology section). Table 1 lists the statistics for each stage.

**Table 1:** Distribution of significant genes and significant DNA methylated genes across the four stages of LUAD

**Stage**	**Number of normal samples**	**Number of disease samples**	**Significant genes**	**DNA methylated genes**	
				**Hyper**	**Hypo**
I	2	9	15994	67	5
II	7	14	16275	20	73
III	5	11	14688	110	60
IV	2	6	14814	0	0

**Figure 1:**
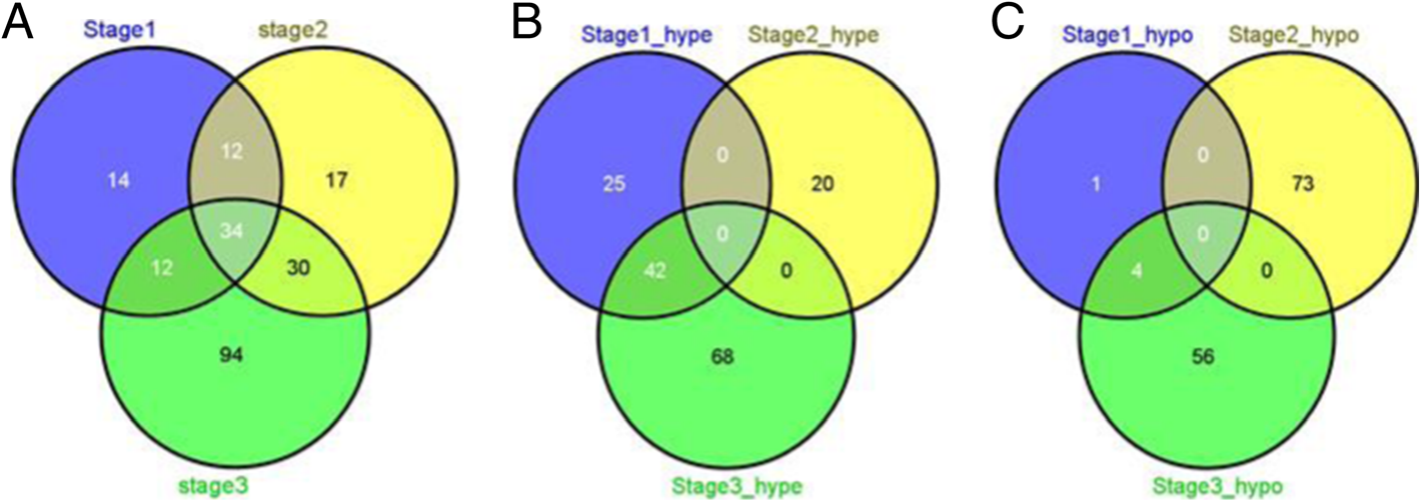
(A) Venn diagram of all DNA methylated genes across stages; (B) Venn diagram of hypermethylated genes across stages; (C) Venn diagram of hypomethylated genes across stages.

The Significant DNA methylated genes were compared across stages as shown in Figure 1A. From the Venn diagrams of Figure 1A it can be seen that the maximum number of Significant DNA methylated genes were identified in Stage III and minimum in Stage I.

As shown in Figure 1A, there were 34 common Significant DNA methylated genes across all stages. Additionally 46 common Significant DNA methylated genes were identified between Stages I and III, and 64 were identified between Stages II and III. Figure 1B and 1C shows the distribution of hypermethylated and hypomethylated genes across stages. Of the 46 common Significant DNA methylated genes between Stages I and III, 42 were was hypermethylated in both stages. On comparing with Stage II, it was observed 36 hypermethylated genes in Stage I were hypomethylated in Stage II and 25 of these were then hypermethylated in Stage III. From Figure 1C it can be stated that maximum number of hypomethylated genes were identified in Stage II. Similar to hypermethylated genes, no genes maintained the same hypomethylation profile across the three stages. Of the four common hypomethylated genes between Stages I and III, two were identified as hypermethylated in Stage II. This suggests that genes in Stage II may have different patterns from those of Stages I and III. Table 2 lists the common Significant DNA methylated genes across and between stages.

**Table 2:** Common DNA methylated genes across stages

***Stages***	***DNA methylated genes***	
	***Number***	***List of genes***
Common DNA methylated genes across the three stages	34	*AJAP1*, *ATP8A2*, *CCDC140*, *CNTP2*, *CYYR1*, *EVX1*, *FERD3L*, *FOXG1*, *GRIK3*, *GRM6*, *HAND2*, *HOXA9*, *HOXB4*, *HOXD4*, *HOXD9*, *HOXD12*, *INPP5B*, *OTX2*, *KRTAP8-1*, *MMP26*, *PHOX2A*, *PLEKHA6*, *POU4F2*, *PRAC, PRKCB*, *PTGDR*, *REG3A*, *SIX6*, *SLC6A2*, *SPAG6*, *TBX20*, *TLX3*, *TMEM130*, *ZNF560*
Common DNA methylated genes across Stage I & II	12	*ADCY4, BHMT, C12orf34, CDO1, LVRN, LY96, MSC, PCDHGA12, POU3F3, ZNF154, ZNF577, IHH*
Common DNA methylated genes across Stage II & III	30	*BARHL2, C10orf81, CCDC140, DEFB119, DIO3, FAM135B, FAM83A, GRIK2, HOXA4, HOXD10, HS3ST2, KCNS2, KRTAP15-1, LEP, LHX1, LYPD5, MAGEB6, NEUROG1, NKX6-2, OR5I1, SERPINB5, SPHKAP, TAL1, TBX4, TBX5, TCN1, TMEM132D, VSX1, ZNF454, IGKV7-3*
Common *hypermethylated* genes across Stage I and Stage III	42	*AJAP1*, *ATP8A2*, *CCDC140*, *CNTP2*, *CYYR1*, *EVX1*, *FERD3L*, *FOXG1*, *FOXI2*, *GALR1*, GAS7, *GRIK2*, *GRM6*, *HAND2*, *HLA-G*, *HOXA7*, *HOXA9*, *HOXB4*, *HOXD4*, *HOXD8*, *HOXD9*, *HOXD12*, *INPP5B*, *NID2*, *NPY*, *OTX2*, *PAX7*, *PHOX2A*, *PLEKHA6*, *POU4F2*, *PRAC*, *PRKCB*, *PTGDR*, *SIX6*, *SLC6A2*, *SOX17*, *SPAG6*, *TBX20*, *TLX3*, *TMEM130*, *VIPR2*, *ZNF560*
Common *hypomethylated* genes across Stage I and Stage III	4	*CORO6*, *MMP26*, *REG3A, KRTAP8-1*

### Identification of highly scored Significant DNA methylated genes

The significant DNA methylated genes were analyzed and ranked based on their beta-values. Table 3 lists the top 10 hyper/hypomethylated genes across stages in descending order of their beta-values. As shown in Table 3, ten of the top Significant DNA methylated genes in Stage I was Stage common across the three (Table 2). Of these 10, seven: *AJAP1*, *ATP8A2*, *HOXA9*, *PTGDR*, *SIX6*, *TLX3*, *TMEM130* were hypermethylated, and the three: *KRTAP8-1*, *MMP26* and *REG3A* were hypomethylated. Three of the seven (Stage I) genes: *AJAP1*, *TLX3*, *PTGDR* were also identified in Stage III. Interestingly the three top scored hypomethylated genes in Stage I was identified as top scored hypermethylated in Stage II. In addition, some of the top scored DNA methylated genes were common across two stages only (Table 2): *LY96* was the top scored hypomethylated gene and top scored hypermethylated in Stage I and II respectively. While *HOXA4*, *HOXD10*, KRTAP15*-1*, *LEP*, and *NKX6-2* were identified as common across Stage II and III (Table 2). Table 3 also identified unique top scored Significant DNA methylated genes. Tables 2 and 3 have large number of Significant DNA methylated genes common among them.

**Table 3:** Identification of top beta-value scored DNA methylated genes across stages

**Stage**	**Hyper/Hypo**	**Genes in descending order of beta-values (p <0.001)**
I	Hyper	*TLX3* > *NEFM* > *PTGDR* > *AJAP1* > *SIX6* > *HOXA9* > *TMEM130* > *HISTIH3G* > *ATP8A2* > *NID2*
	Hypo	*MMP26* > *KRTAP 8–1* > *REG3A* > *CORO6* > *LY96*
II	Hyper	*LY96* > *C10orf81* > *KRTAP8-1* > *MMP26* > *REG3A* > *DEFB 119* > *NMUR2* > *MAGEB6* > *IGKV7-3* < *KRTAP15-1*
	Hypo	*HTR2C* > *GRIK3* > *CNTP2* > *SPHKAP* > *TMEM132D* > *NEFH* > *LEP* > *ZNF177* > *HOXD10* > *NKX 6-2*
III	Hyper	*TLX3* > *HOXB4* > *AJAP1* > *HOXA4* > *HOXD9* > *PTGDR* > *LYPD5* > *FZD10* > *HOXD12* > *NECAB2*
	Hypo	*CHR6* > *C13orf28* > *TMEM156* > *XDH* > *FGF6* > *IVL* > *G6PC* > *KRTAP8-1* > *C10orf39* > *FCRL3*

### Significant DNA methylated genes in and outside of CpG islands, promoter regions, transcription factors, chromosomes and pathways

**Figure 2:**
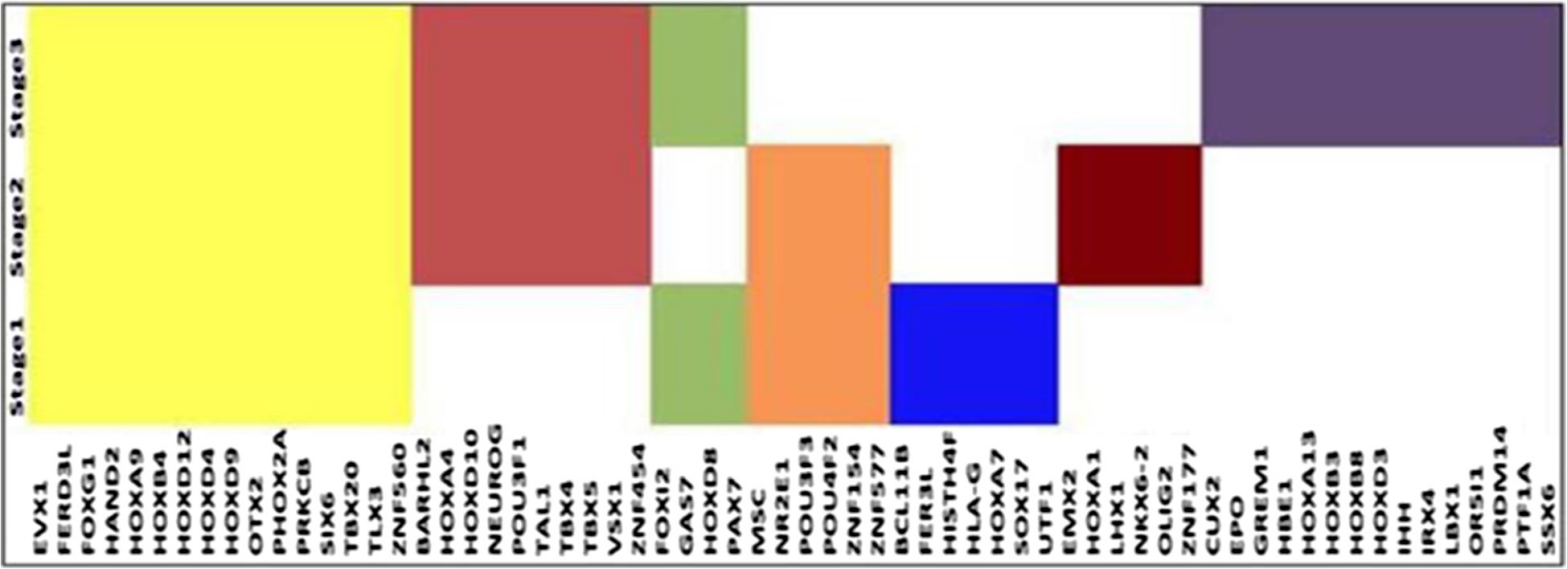
Profile of DNA methylated genes as transcription factors across stages.

The hypermethylated and hypomethylated genes were further analyzed with respect to their methylation inside and outside of the CpG islands. Table 4 gives the profile of this distribution. From this table, hypermethylated genes in Stages I and III and hypomethylated genes in Stage II were mostly identified in TRUE CpG sites. Of the 34 common Significant DNA methylated genes across all stages (see Table 2), 25 were identified in TRUE CpG sites: *AJAP1*, *ATP8A2*, *CYYR1*, *EVX1*, *FERD3L*, *GRIK3*, *GRM6*, *HAND2*, *HOXA9*, *HOXB4*, *HOXD9*, *HOXD4*, *HOXD12*, *OTX2*, *PRAC*, *PHOX2A*, *POU4F2*, *PTGDR*, *SIX6*, *SLC6A2*, *SPAG6*, *TBX20*, *TMEM130*, *TLX3*, and *ZNF560*. These common genes were hypermethylated in Stages I and III respectively but hypomethylated in Stage II. Additionally, nine hypermethylated genes common to Stages I and III (see Table 2) were identified in TRUE CpG sites: *GALR1*, *HLA-G*, *HOXA7*, *HOXD8*, *NID2*, *NPY*, *PAX7*, *SOX17*, and *VIPR2.* The hypomethylated genes which were common to Stages I and II, *REG3A*, *MMP26* and *KRTAP8-1* (see Table 2) were also identified in TRUE CpG sites. The CpG sites were analyzed for their role as promoter sites. This analysis identified 61/72, 80/93 and 141/170 Significant DNA methylated genes across Stage I, II and III respectively in promoter sites. Also, the common Significant DNA methylated genes across all stages and between two stages (see Table 2) were identified in the promoter sites. Methylation of promoter regions in the gene correlate with low or no transcription [33]. Gene Ontology was used to correlate the transcription role of the Significant DNA methylated genes identified in all of the three stages. Recall from Table 1 that there are 72, 93 and 170 Significant DNA methylated genes found in Stages I, II and III respectively of these 65 were identified as TFs. Among the 34 common genes across stages identified in Table 2, 16 were identified as TFs. These 16 TFs were found to be hypermethylated in Stages I and III, and hypomethylated in Stage II. Figure 2 describes the TF distribution profile across the different stages. Analysis of these TFs with respect to their CpG sites also identified all (except *HBE1, HOXD10*, *OR51*) mapped to the TRUE CpG sites.

**Table 4:** Distribution of hyper and hypo-methylated genes in CpG islands

**Stage**	**Hypermethylated**		**Hypomethylated**	
	**True**	**False**	**True**	**False**
I	53	4	1	4
II	4	14	60	4
III	88	7	4	45

**Figure 3:**
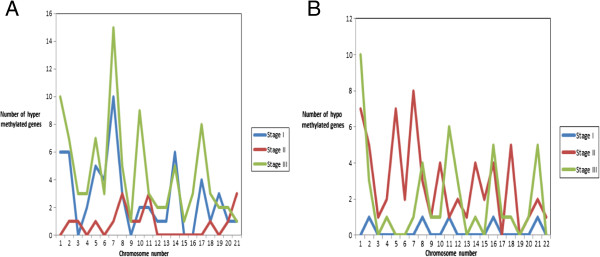
**Profile of methylated genes. A** Distribution of hypermethylated genes across chromosomes in different stages. **B** Distribution of hypomethylated genes across chromosomes in different stages.

Figure 3A and 3B show the chromosome profile of Significant DNA methylated genes with respect to their hyper and hypomethylation. As shown in Figure 3A for Stage I the maximum number of hypermethylated genes were present on chromosome 7 while no hypermethylated genes were present on chromosomes 3, 9, 15 and 16. The Stage I genes identified on chromosome 7: *EVX1*, *FERD3L*, *HOXA7*, *HOXA9*, *NPY*, *TBX20* and *TMEM130* were common in Stages I and III; five of these were common across all stages (see Table 2). For Stage II, the maximum number of hypermethylated genes was present on chromosome 8: *MX2*, *KRTAP8-1* and *KRTAP15-1*. Of these three, *KRTAP8-1* was common across all the stages, and *KRTAP15-1* common across Stages II and III (see Table 2). In Stage III, all chromosomes had atleast one gene identified as hypermethylated. As with Stage I, in Stage III also the maximum number of hypermethylated genes was identified on chromosome 7: *CFTR*, *DGKI, EPO*, *EVX1*, *FERD3L*, *HOXA4*, *HOXA7*, *HOXA9*, *HOXA13, LEP*, *NPTX2*, *NPY*, *TBX20*, *TMEM130* and *VIPR2*. Of these genes five were common across all stages, three were common to Stages I and III; two were common to Stages II and III; and two in only Stage III (see Table 2).

Figure 3B shows Stage II has a maximum number of hypomethylated genes on chromosome 7 also no hypomethylated genes were identified on chromosomes 18 and 19 for this stage. For Stage I, only one hypomethylated gene was identified in chromosomes 2, 8, 11, 17, 21. For Stage III, maximum number of hypomethylated genes was present on chromosome 1: *CD1B*, *FCRL3*, *FLG*, *IVL*, *LCE1F*, *LCE2B*, *LCE3D*, *LCE4A*, *SPTA1* and *ZP4*. Also, these 10 hypomethylated genes on chromosome 1 were unique to Stage III.

Additional file 2 shows the pathway distribution of Significant DNA methylated genes across stages. This distribution depicts difference and commonality across stages in terms of pathways. The pathways associated with common Significant DNA methylated genes given in Table 2 across all stages were Inositol phosphate metabolism, Neuroactive ligand-receptor interaction, Phosphatidylinositol signaling system and P53 signaling. The pathways associated with common Significant DNA methylated genes (Table 2) across Stages I and II were Glycine, serine and threonine metabolism, Melanogenesis, Taurine and hypotaurine metabolism, P.E.coli infection, Shigellosis, Toll-like receptor, calcium signaling pathway and GnRH signaling pathway. The pathways associated with common Significant DNA methylated genes (Table 2) across Stages II, and III was Adipocytokine signaling pathway, Cytokine-cytokine receptor interaction, JAK-STAT signaling pathway, P53 signaling pathway. The pathways associated with common Significant DNA methylated genes across (Table 2) Stages I and III were Antigen processing, Cell adhesion molecules, Natural killer cell mediated cytotoxicity, Type I diabetes mellitus. In addition, focal adhesion pathway was associated with Stage II genes and Hedgehog pathway with Stage III genes.

### Network construction and analysis

A systems biology approach was developed to understand the Significant DNA methylated genes and Significant expressed genes in each stage. The interactions for the Significant DNA methylated genes and Significant expressed genes were identified using BioGRID [34] and stage-specific networks were constructed. Table 5 shows the number of interactions of Significant DNA methylated genes across the three stages. In each stage, the interactions of Significant DNA methylated genes were analyzed with respect to Significant expressed genes. This analysis showed that Significant DNA methylated genes have interactions among themselves, with Significant expressed genes and additional genes present in BioGRID [34]. These additional genes were analyzed for their expression in all the stages to determine if DNA methylation affected their expression. These interactions were termed as “missing links”, and the additional genes as “novel genes”. Table 6 gives the profile of the missing links and novel genes. Analysis of the 27 novel genes in Stage I for their significance in other stages indicated six of them in Stage II: *ANXA7*, *APBB1IP*, *MDK*, *PFDN1*, *TINF2, TLE2;* three in Stage III: *CUL5*, *CTNNB1* and *SQSTM1* and six in Stages II and III: CALM1, *CTNNB1*, c-*JUN*, *SMAD1*, *TINF2*. Of the 33 novel genes in Stage II, two were associated in Stage III: *A2M* and *CTNNB1*I; and ten genes in Stages I and III: *FOXA2*, *HK3*, *NCF1*, *NRIP1*, *PDLIM1*, *SP1*, *SUMO1*, *TCF4*, *TLR4*, and *TNN*. Analyses of the 83 novel genes in Stage III found three in Stage I: *ELN*, *FAS* and *TEX11*; seven in Stage II: *ANXA7*, *APBB1IP*, *MDK*, *PFDN1*, *STAT3*, *TLE2*, *UBE2B* and 34 in Stages I and II:*BCR*, *DLG3m, DLG4*, *EGFR*, *DSP*, *MAFF*, *PICK1* etc. Table 7 shows the profile of interaction of Significant DNA methylated genes identified in Table 2 and Table 3 with novel genes analyzed in this paragraph (given in Table 6).

**Table 5:** DNA methylated gene interactions across stages

**Stage**	**Num. of DNA methylated genes**	**Num. of interactions**
I	72	228
II	93	273
III	170	660

**Table 6:** Novel genes (Missing Link-methodology) discovered using BioGRID

**Stage**	**Missing links**	**Novel genes**	**Num. of DNA methylated genes**	**Num. of novel genes & stages where these are identified**
I	27	27	16	6 (Stage II, III), 6 (Stage II),3 (Stage III)
II	43	33	25	10 (Stage I, III), 2 (Stage III)
III	132	83	32	34 (Stage I, II), 3 (Stage I), 7 (Stage II)

**Table 7:** Analysis of DNA methylated genes interacting with novel genes

**DNA methylated genes**	**Stages**		
	**I**	**II**	**III**
*Common DNA methylated genes*			
*AJAP1*	√	√	√
*FOXG1*	√	√	√
*GRIK3*		√	√
*HAND2*			√
*HOXD4*	√	√	√
*PHOX2A*	√		
*PRKCB*	√	√	√
*TLX3*		√	
*DNA methylated genes common with Stage I & II*			
*LY96*	√	√	
*MSC*		√	
*Hypermethylated genes common with Stage I & III*			
*GAS7*	√		√
*GRIK3*	√		
*HLA-G*			√
*HOXA9*			√
*HOXD8*			
*HOXD12*			√
*NID2*			
*TLX3*			√
*DNA methylated genes common with Stage II & III*			
*SERPINB5*		√	√
*TAL1*			√
*Top scored DNA methylated gene*			
*IVL*			√
*NEFM*	√		
*TLX3*	√		√

**Figure 4:**
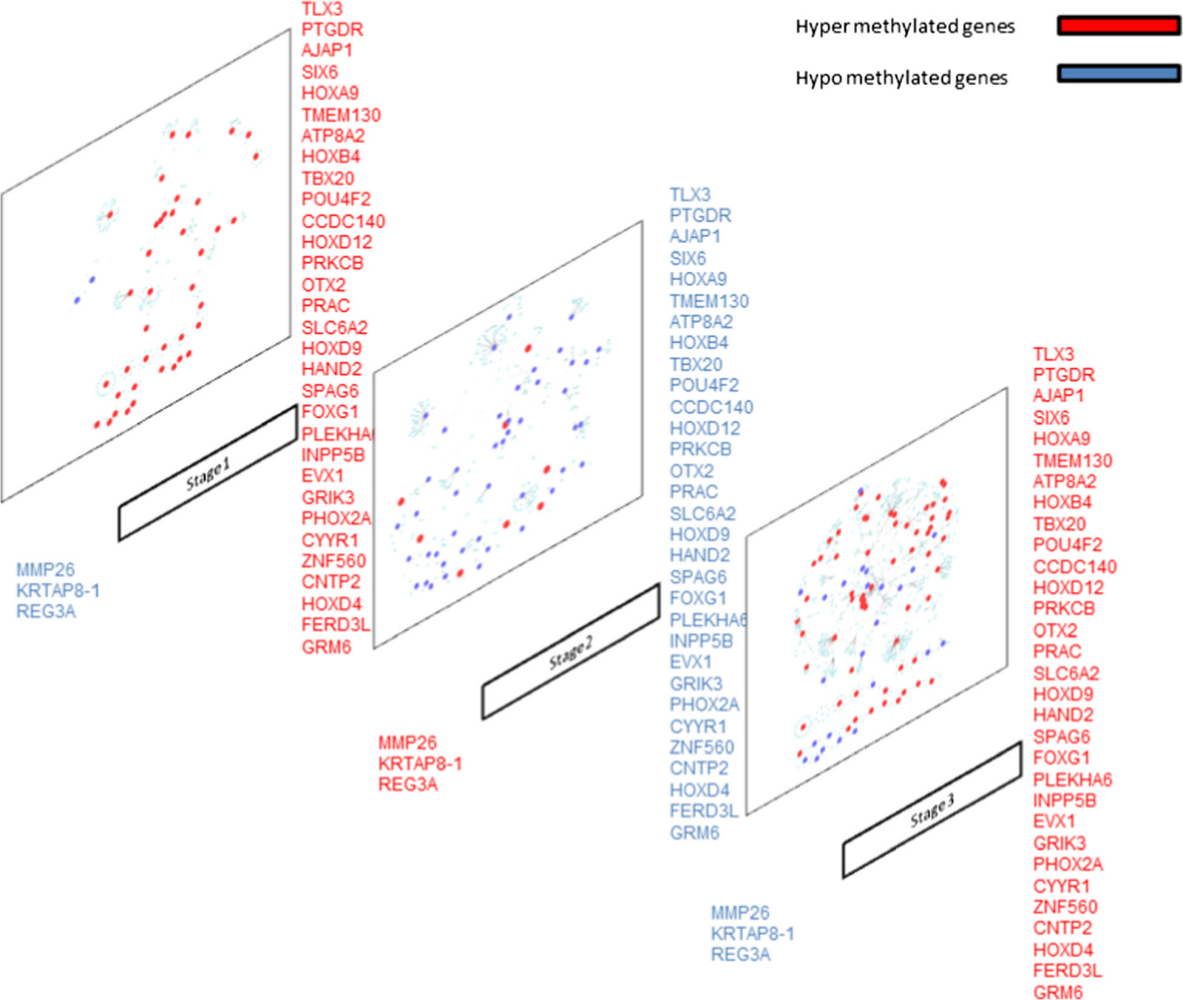
Stage-wise interactions of DNA-methylated genes with significant genes.

Figure 4 shows the stage-specific networks of Significant DNA methylated genes. From this figure, it can be seen that Stage III networks were more connected and dense as compared to other two stage networks. This suggests heterogeneity of LUAD network across stages. To compare stage-specific networks, subnetworks of Significant DNA methylated genes were identified and analyzed. SEED and expand algorithm (described in methodology) was used to identify these subnetworks. Additional file 3 lists the number of subnetworks with respect to the pathway class. These subnetworks were overlapping as the genes in them belonged to different pathway class. Additional file 3 shows that the number of subnetworks drastically increases from size four to size five in most of the stages, making it an NP-hard problem. This sharp increase in the number of subnetwork suggests that though the DNA methylated gene is not directly connected to a hub node, its interaction path has a hub node. This further indicates that a DNA methylated gene can influence the whole network of a given stage. Table 8 lists the subnetworks with greater number of connections identified in all three stages. As shown in Table 8, *UBC* and *CUL1* were identified as hub gene across the three stages and their connectivity profile changes with pathway class. The other hub genes (number of connections) identified in Stages I and III (not shown in the Table 8) were: *SIRT7* (6), *CDK2* (5), *PMS2* (4), *SUMO2* (3), *SMAD3* (7), *SMAD4* (5), and *SMAD2* (4). The analysis also identified *LY96* subnetwork in Stage I consisting of the hub gene *TLR4* interacting with seven other genes. Though *LY96* was also identified in Stage II, the comparative subnetwork was smaller, and this gene was not identified at all in Stage III. *HLA-*G was present in Stage I but not in Stage II; therefore its subnetworks were missing. In Stage II and III, *c-Jun* a TF was identified as a hub gene. *PHOX2A* was the Significant DNA methylated gene associated with *c-Jun* in both stages. There was similarity across common genes (see Table 2) with table 8, depicting that subnetworks constructed out of common genes across or between two stages can be of significance to LUAD. The size four subnetworks were further compared across the stages to understand their commonality and uniqueness (Additional file 4). This size four subnetworks were analyzed for their common Significant DNA methylated genes. The common Significant DNA methylated genes in this size four subnetworks were *FOXG1*, and *PHOX2A (*see in Table 2 also) and significant expressed genes were: *FOXH1*, *FOXO3*, *HAND2*, *MYC*, *RB1, SMAD2*, *SMAD3*, *SMAD4*, and *TP53.* On analysis of genes in these subnetworks with respect to their pathway classes found some of them to be very specific to a given pathway class. A highly conserved common subnetwork of GRIK2, *GRIK3*, *GRIK5*, and *GRID2* was identified across all stages belonging to the other pathway class. Of these *GRIK3* was Significant DNA methylated in all the three stages (Table 2) and *GRIK2* in Stages I and III (Table 2).

**Table 8:** Analysis of hub genes in the DNA methylated subnetworks of size 4

**Stage**	**Subnetwork**	**Connectivity profile of the hub node across pathways**			
		**Cancer**	**Lung cancer**	**Signaling**	**Metabolic + others**
I	(i) PHOX2A*:HAND2*:*PPP2R5D*: ***UBC***	23	13	71	288
	(ii) HLA-G*:*COPB1*:*TRIM37*: ***UBC***				
	(iii) LY96*:*TLR4*:*SIGIRR*: ***UBC***				
	(iv) HLA-G*:*COPB1*:*UBC*: ***CUL1***	21	14	35	114
	*(v*) FOXG1*:*FOXH1*:*SMAD3*: ***CUL1***				
	(vi) HLA-G*:*COPB1*:*UBC*: ***SKP2***	19	10	17	38
II	(i) PHOX2A*:*c-JUN*:*SUMO3*: ***UBC***	23	14	74	254
	(ii) TAL1*:*HDAC1*:*IRF5*: ***UBC***				
	(iii) PRKCB*:*HIST1H3I*:*CUL4A*: ***CUL1***	20	14	74	253
III	(*i*) PHOX2A*:*c-JUN*:*SUMO3*: ***UBC***	22	10	64	235
	(*ii*) PHOX2A*:HAND2*:*PPP2R5D*: ***UBC***				
	(iii) PRKCB*:*HIST1H3I*:*CUL4A*: ***CUL1***	20	13	35	102

*: identified in Table2.

**Figure 5:**
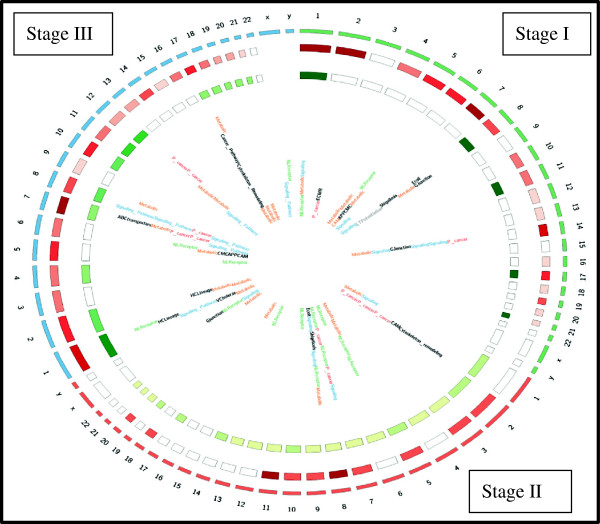
**Circos image showing the stage-wise distribution of hypermethylated, hypomethylated genes and pathway class in the chromosomes.** Outer circle represents the chromosomes, the first inner circle represents the hypermethylated genes in each chromosome, and the second inner circle represents the hypomethylated genes on each chromosome and inside is the pathway class to which the gene on the respective chromosome belongs.

Analysis of these subnetworks is an NP-hard problem because these are large open subnetworks. To reduce the complexity, the subnetworks were ranked based on their *NodeStrength* and *EdgeStrength* as given in methodology section. The top ranked, size four subnetworks of each stage (Table 8 and Additional file 3) were propagated and compared to identify the largest conserved subnetworks across the stages. This analysis identified a subnetwork of size 11 with seven conserved genes: *UBC*, KRAS, *PIK3CA*, *PIK3R3*, *RAF1*, *BRAF*, *RAP1A* (Additional file 5). The g: Profiler tool was used for the enrichment analysis on the top ranked subnetwork given in Table 9[35,36]. This analysis showed that these subnetworks to be enriched with common genes across stages (shown in Table 2), indicating that commonality across stages of LUAD can be critical in identifying the target genes. Figure 5 gives the Circos image of the number of hypomethylated and hypermethylated genes and pathways class across chromosomes for each stages of LUAD.

**Table 9:** Enrichment analysis of the top scored subnetworks

***Stage***	***Biological Process***	***p-value***	***Genes in subnetworks***
Common across all Stages	Activation of MAPKK activity	1.12E-03	PHOX2A*, HAND2*, *PPP2R5D*, *UBC*, *KRAS*, *PIK3CA*, *PIK3R3*, *RAF1*, *BRAF*, *RAP1A*
I & II	Co-SMAD binding	1.2E-05	FOXG1*, *FOXH1*, *SMAD2*, *SMAD1*, *MED15*, *UBC*, *KRAS*, *PIK3CA*, *PIK3R3*, *RAF1*, *BRAF*, *RAP1A*
II & III	Nerve growth factor receptor signaling pathways	2.21E-04	HOXD4*, *INPP5B*, *SLC6A2*, *STX1A*, *VAMP1*, *UBC*, *KRAS*, *PIK3CA*, *PIK3R3*, *RAF1*, *BRAF*, *RAPIA*
I & III	Positive regulation of peptidyl-serine phosphorylation	1.42E-03	NPY*, *NPY1R*, *LSM7*, *NR1H2*, *RMI1*, *UBC*, *KRAS*, *PIK3CA*, *PIK3R3*, *RAF1*, *BRAF*
I	Transmembrane receptor protein tyrosine kinase signaling pathway	1.87E-03	HLA-G*, *COPB1*, *UBC*, *KRAS*, *PIK3CA*, *PIK3R3*, *RAF1*, *BRAF*, *RAP1A*
II	DNA helicase complex	6.8E-05	SERPINB5*, *UCHL5*, *ACTR8*, *ACTR5*, *UBC*, *KRAS*, *PIK3CA*, *PIK3R3*, *RAF1*, *BRAF*
III	Nerve growth factor receptor signaling pathway	5.5E-04	HOXB4*, *CREBBP*, *KLF13*, *UBC*, *KRAS*, *PIK3CA*, *PIK3R3*, *RAF1*, *BRAF*, *RAP1A*

*: commonality with Table 2.

**Figure 6:**
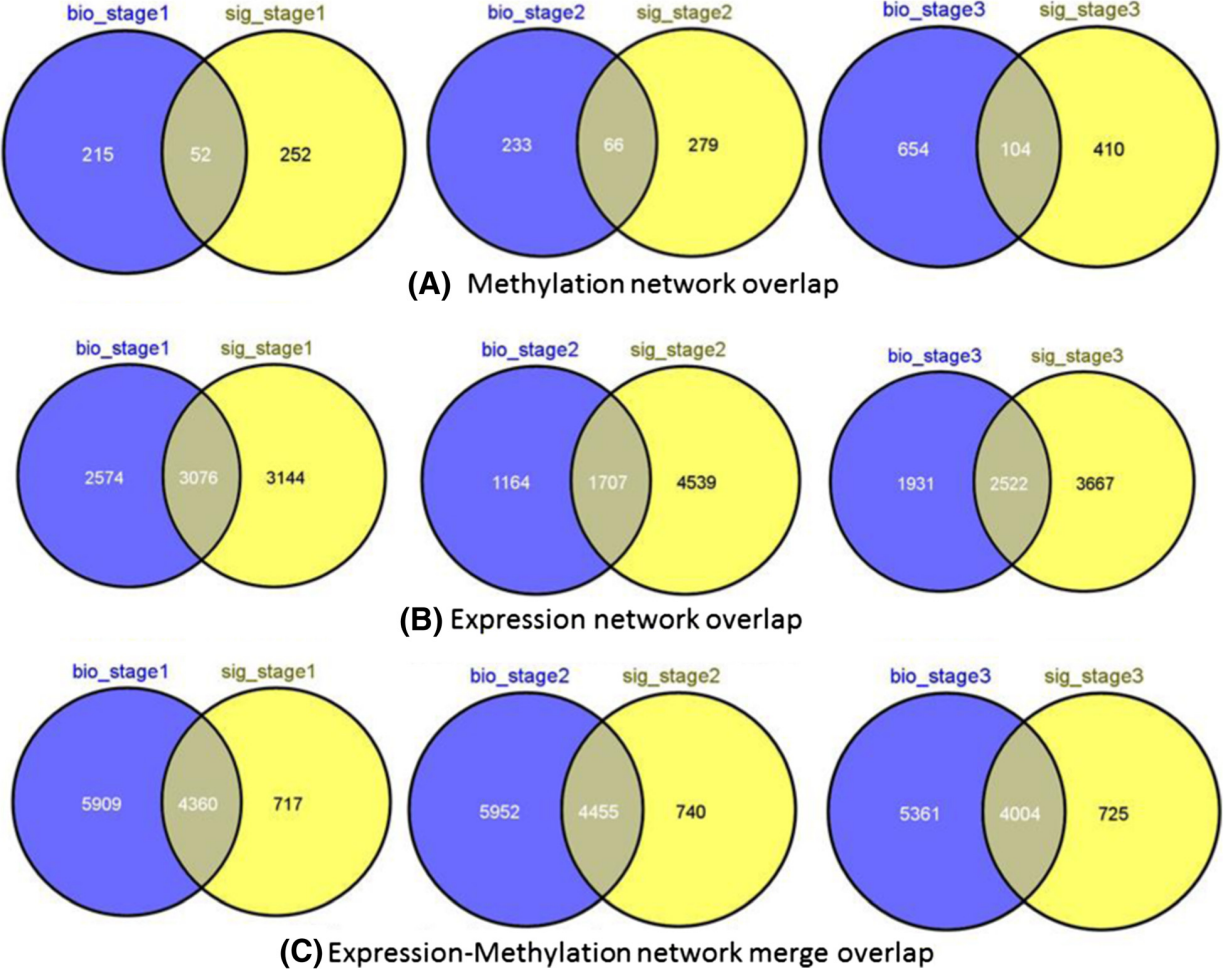
**Comparison of different types of networks obtained from BioGRID and manually curated signaling network.** The “bio_stage” refers to BioGRID and “sig_stage” refers to signaling network. The Venn diagram depicts the overlap of genes between the two networks obtained from the two different sources, BioGRID and Signaling network. **A)** Venn diagram of the methylated genes for Stage I, Stage II, and Stage III. **(B)** Venn diagram of expression genes for Stage I, Stage II, and Stage III. **(C)** Venn diagram of methylated-expression genes for Stage I, Stage II, and Stage III.

Figure 6 and Table 10 show the comparison results for the interaction networks from BioGRID and from the manually curated signaling network. As shown in the table and Venn diagram, there is a minimal proportion of overlap between the methylation and expression networks obtained from multiple sources (see Figure 6(A) & (B)). However, this overlap improves after applying the methylation-expression network integration criteria mentioned in methodology section (see Figure 6(C)). In addition, there is a significant amount of commonality in the subnetworks extracted from networks for BioGRID and the manually curated signaling network. The same set of conserved genes (*KRAS*, *PIK3CA*, *PIK3R3*, *RAF1*, *BRAF*, *and RAP1A*) was obtained in these networks except for *UBC*, which is missing from the resulting subnetwork of signaling network.

**Table 10:** Percentage of genes overlapping from signaling network with BioGRID network

	**Stage 1 (%)**	**Stage 2 (%)**	**Stage 3 (%)**
**Methylation network**	17.1	19.1	20.2
**Expression network**	49.45	27.32	40.75
**Expression-Methylation merged network**	85.9	85.75	84.60

## Discussion

### Distribution and understanding of Significant DNA methylated genes across stages

According to Table 1 and Figure 1, the maximum number of Significant DNA methylated genes were identified for Stage III followed by Stage II and then Stage I. None of the genes in Stage IV met the filtering criteria; therefore, no genes were identified as DNA methylated. From Table 1, it can be seen that hypermethylated genes were more prevalent in Stages I and III than in Stage II. Though this study identified 34 common Significant DNA methylated genes (see Table 2) across the three stages, most of them have not been reported previously in LUAD. The HOX genes that were common across the three stages are grouped into four HOX families, A, B, C, and D; equivalent numbered HOX genes (HOXA9, HOXB9) in each family groups (A, B, C, D) are paralogues. The analysis found *HOXA4*, *HOXA9*, *HOXB4*, *HOXD9*, and *HOXD12* genes with high methylation value, suggesting these genes play an important role in all stages of LUAD. These genes are known to be involved in cell proliferation while preventing apoptosis and helping in survival [37]. Dysregulated behavior of HOX genes has been observed in ovarian cancer [38]. Early stage *HOXA9* methylation has been identified in lung cancer and used in early detection and prognosis [39,40]. Our analysis found HOX genes in all stages, with hypermethylation in Stages I and III, hypomethylation in Stage II. While no previous studies have associated the profile of HOX genes with stages, though re-appearance was identified and our analysis demonstrated this aspect. Another gene identified by our analysis across all three stages was *PTGDR,* which was highly hypermethylated in Stages I and III (Table 3). *PTGDR* has been negatively correlated with smoking [41] and methylated in colon cancer [42], however, prior studies have not investigated its role in LUAD. *POU4F2* and *TLX3* were identified in all three stages, and *TLX3* was highly methylated in Stages I and III (Table 3). Previous studies have found them as methylated in leukemia and breast cancer respectively [43,44] but not in LUAD. Overexpression of *TBX20*, which was also identified in this study (see Table 3), has been reported in lung cancer [45]. *EVX1* and *OTX2* (see Table 2) were identified as methylated in NSCLC and lung cancer [46,47]. *MMP26* has been associated with tumor development, invasion and metastasis of NSCLC but its methylation profile was not reported [48], our analysis showed it to be highly hypomethylated in Stage II (Table 3). There was no literature evidence about *KPTAP8-1*, *REG2A*, and *SLX6* for their significance or methylation in lung cancer.

Of the 12 common Significant DNA methylated genes common to Stages I and II, *LY96* has been previously associated with lung cancer [49]; *ZNF577* and *LVRN* have been identified as methylated in lung cancer [47] and renal carcinoma, but not in LUAD [50]. *LY96* was highly hypomethylated in Stage I and hypermethylated in Stage II (as shown in Table 3), suggesting further investigation into its role in LUAD.

Of the 30 common Significant DNA methylated genes across Stages II and III, *GRIK2* and *NEUROG1* have been previously reported being DNA methylated biomarker for lung squamous cell carcinoma [47], and a Stage I biomarker in lung cancer [46] respectively. However, re-appearance of *NEUROG1* in Stage III has not been previously reported. *SERPINB5* and *TAL1* have been identified as methylated in NSCLC [30,46,51]. *LEP* has been reported as biomarkers in breast cancer [52], though not in lung cancer. The other highly hypermethylated genes across Stages II and III identified in this study (as shown in Table 3) were *AJAP1*, *HOXB4*, *MMP26*, *NMUR2*, *REG3A*, *TLX3*, etc. and hypomethylated genes were *FCRL3*, *GRIK3*, *HTR2C*, *IVL*, *NKX6-2*, etc. Literature validation of these genes with respect to their importance in LUAD and other cancers found *NMUR2* to be overexpressed in pancreatic cancer [53], *AJAP1* epigenetically silenced in Glioblastoma [54]. Also, *AJAP1* was correlated with susceptibility in lung cancer [55]. *GRIK3* was correlated with breast cancer and being considered as diagnostic for lung cancer [56]. Not much literature evidence has been reported for the presence of *VSX1*, *NKX 6–2* in cancer or their methylation.

Of the 42 common Significant hypermethylated genes unique to Stages I and III, *GALR1*, *NID2* have been identified as highly methylated in NSCLC [46,55], *PAX7* has been identified in lung cancer but not reported with methylation [57], though *PAX* family genes have been previously reported being methylated in cancer [58]. Recent studies have reported *SOX17* methylation in lung cancer [59], but not at the stage level. Additionally low expression of *GAS7* has been reported in lung squamous cell carcinoma suggesting its importance as marker [60], but this gene has not been previously found to be methylated in LUAD but reported as methylated in colorectal cancer [61].

Of the four Significant hypomethylated genes in Stage I and III, in addition to *KRATAP8-1* and *MMP26* which were analyzed in the above section, *CORO6* was also hypomethylated and has been reported as an epigenetic gene in renal cell carcinoma but not in LUAD [62]. However, not much detail for *REG3A* with regards to its presence in LUAD was available.

In addition to genes that were found to be common across all or two stages, several genes were unique to one stage only, and these need further evaluation. From Stage I, NEFM has been reported as a biomarker and as a methylated gene in cancer [63,64], however, it was not reported in LUAD. From Stage III, *IVL* has been identified as overexpressed in cancer [65] but not in LUAD. Overall our methodology identified both known and novel DNA methylated genes that were significant across all three stages of LUAD. Also, our analysis found that most of the DNA methylated genes that were common across all stages were highly methylated in the respective stages (Table 2 and Table 3), and many of them were reported as oncogenes.

### Distribution of the Significant DNA methylated genes in and outside of CpG islands, promoter regions, transcription factors, chromosomes and pathways

Our initial distribution analysis found that most of the Significant DNA methylated genes across the three stages were present in the TRUE CpG sites (as shown in Table 4), stating the role of these sites in methylation. In Stage III, 45 hypomethylated genes were present in FALSE CpG islands. The false sites could be further validated using other databases or clinical features. The analytic procedure in this analysis identified 30 of the 34 common Significant DNA methylated genes (found in Table 2) and 79 unique hypermethylated Stage III genes in the promoter regions. This correlation of the promoter region with common Significant DNA methylated genes further demonstrates that the genes with higher CpG islands in the promoter region were methylated across the stages of LUAD. Therefore, further analysis can be done to better understand these promoter regions functionally with respect to their conservation (motifs) as these can be co-regulated.

DNA methylation is closely linked with gene regulation, particularly with transcriptional activity. It has been reported that DNA methylation can prevent gene activation and restrict expression for correct developmental stage [66]. It can also interfere with binding of TFs by changing the recognition sites involving cytosine [67]. Most TFs require CpG-rich sites to bind to DNA and methylation of these sites might interfere with the bindings. This study identified TFs in all three stages and these TFs were associated with TRUE CpG sites. As TFs have been identified as potential biomarkers for different diseases, the unique TFs identified for each stage were analyzed for their significance in LUAD using the literature. Of the 16 TFs common across the stages, four (*EVX1*, *HOXA9*, *OTX2*, *TLX3*) have already been discussed in earlier sections. Table 11 lists the significance of the remaining few common and unique TFs across the stages with respect to their association in lung cancer, other cancer (not lung) and/or prognostic value. From this table, it can be seen that almost all the TFs are considered as prognostic markers for lung cancer. Our study identified these TFs as epigenetically modified across the stages of LUAD, and, given their significance in cancers (other or lung), they could be considered for future studies as potential targets for LUAD.

**Table 11:** Significance of transcription factors in LUAD

**Stage/DNA methylated genes identified as TFs**	**Significance**
***Common across Stages (Table*** 2 ***)***	
*FOXG1*	Cell adhesion, Growth and invasion of lung cancer [68] and is prognostic marker in bladder cancer [69]
*HAND2*	Identified in early stage in squamous cell carcinoma but not in adenocarcinoma [70]
*HOXB4*	Overexpression identified in ovarian cancer though HOX genes are reported in lung cancer [71]
*PHOX2A*	Abnormal methylation in NSCLC [72]
***Unique TFs in Stage I (total 6)***	
*HLA-G*	Potential biomarker in lung cancer [73]
*BCL11B*	A new therapeutic for T-cell malignancies but direct correlation with lung cancer not available [74,75]
*UTF1*	Not yet correlated in lung cancer but a prognostic in cervical cancer [76]
***Unique TFs in Stage II (total 6)***	
*EMX2*	Is associated with WNT signaling pathway and its down-regulation is associated with methylation of promoter region in lung cancer suggesting it as novel suppressor gene for human lung cancer [77]
*NKX6-2*	Identified as methylated in lung cancer [40] and identified as target in Pancreatic cancer [78]
*OLIG2*	Identified in lung cancer but not study as target [79]
*ZNF577*	Identified as methylated in various cancers like breast and oropharyngeal squamous cell carcinoma, very recently in lung cancer [80]. Not much is reported with respect to its prognostic value.
***Unique TFs in Stage III (total 13)***	
*EPO*	Is the key regulator in the production of *RBC*, methylation of the promoter section of EPO is identified in many cancers including lung, breast liver etc. [81] though its prognostic value efficiency is not reported.
*GERM1*	Hypermethylation of *GREM1* is identified for prognostic significance in renal cell carcinoma [82], so far not in lung cancer.
*IRX1*	Methylation is identified in lung cancer [83] but needs to be studied for prognostic markers.

Earlier studies have reported chromosome 6 and 15 to play an important role in lung cancer [84,85] also certain chromosomal regions were more hypermethylated [15]. On mapping, the hypermethylated and hypomethylated genes to their respective chromosomes, it was observed that some chromosomes were common across all the three stages (Figure 3A and Figure 3B). Chromosome 7 was identified in all three stages, with the maximum number of hypermethylated genes in Stages I and III. Six common Significant DNA methylated genes of Table 2 were identified on chromosome 7 including the *HOX* genes which cluster on chromosomes 2, 7, 12 and 17. Our analysis found chromosome 7 to be highly epigenetically modified. Some of the other methylated genes located on this chromosome found in the Stages I and III were *EVX1*, *FERD3L*, *NPY*, *TBX20*, and *VIPR2. NPY* was found to be highly expressed in prostate carcinomas [86] while the significance of the others genes was discussed in the previous section. Another Significant DNA methylated gene common across Stages II and III (Table 2) identified on chromosome 7 was *LEP*. This gene is known to be associated with advanced lung cancer (http://www.ncbi.nlm.nih.gov/gene/3952). Genes on chromosome 7 have also been reported to associate with different cancers including gastric cancer, and prostate cancer [87,88]. Chromosome 7 genes *AKT* and *PTEN* are used as prognostic markers for NSCLC [89], suggesting that chromosome 7 genes that have been identified across stages of LUAD as methylated can be considered for prognostic significance in LUAD. Our analysis also found Chromosomes 17 and 14 to be associated with a large number of hypermethylated genes in Stages I and III. Chromosome 17 has been previously studied and associated with NSCLC [90]. Chromosome 14 has been associated with genetic variation in lung cancer [91]. In Stage III chromosome, 10 was identified with nine hypermethylated genes and these were: *LBX1*, *NKX6-2*, *PTF1A*, *SLC18A3*, *SORC3*, *SPAG6*, *C10orf26*, and *C10orf82*. The ladybird homeobox 1 (*LBX1*) gene has been associated with the breakpoint regions involved in T-cell leukemia [92] and methylated in prostate cancer [93]. However, not much has been studied and reported about *LBX1* methylation and association in lung cancer. Similarly *NKX6-2* is a methylated biomarker for bladder cancer but its importance and methylation has not been studied in LUAD [94].

The analysis found that chromosomes with the highest number of hypermethylated genes in Stage I also had the highest number of hypomethylated genes in Stage II and eventually have the highest number of hypermethylated genes in Stage III (Figure 3A and Figure 3B). Also in Stage II a large number of hypomethylated genes were identified in almost all the chromosomes. This observation suggests a distinct methylation pattern across the three stages of LUAD, and since methylated genes are present on certain chromosomes in cancer, further indicating that epigenetics plays an important role in LUAD.

The pathway analysis depicted the onset of different epigenetically modified pathways across stages. The common signaling pathway identified across the three stages shown in Additional file 2 was: Adipocytokine signaling and Phosphatidylinositol signaling; across Stages I and II were: Toll-like receptor, Calcium signaling, GnRH signaling pathway; across Stages II and III were: *JAK*-*STAT* and *P53* signaling pathways. It has been reported that genes silenced due to promoter methylation were mostly tumor suppressor genes [15], and silencing of these genes can eventually affect all the pathways especially cell cycle, DNA repair genes, apoptosis, signaling etc., which could lead to tumor progression. An example of this propagation was gene *LY96* which was identified in our analysis as hypermethylated in Stage I and hypomethylated in Stage II (Table 2). In Stage I, the interacting genes were *TLR2*, *TLR4,* while in Stage II its interacting genes were *TLR2*, *CALM1* and *UBC. LY96* encodes *MD2* a molecule important for the activation of *TLR4*, which promotes survival [95] and Toll-like pathways connect to the immune system [96]. DNA methylation of *LY96* might prevent the activation of *TLR4* in Stage II, which in turn would affect the activation of Toll-like pathway. Since cancer cells evade the immune system, reversing the epigenetic behavior of *LY96* needs to be further evaluated as it could result in the activation of TLRs which would be beneficial. Similar analysis can be carried out for the other pathways that are common across stages as these have also been identified as important cancer signaling pathways [74,97]. In addition, the focal adhesion pathway associated with Stage II has been reported to be involved with multiple signaling events in lung cancer, suggesting that methylation of this pathway might also affect the signaling pathways [98]. Hedgehog pathways in Stage III have been identified as a subset of NSCLC and are being investigated for clinical trials [99]. Our analysis also found that metabolic pathways are co-related with the DNA methylated genes in each stage, underscoring the fact that methylation affects important pathways in LUAD. Our analysis also depicted the early and late methylated affected pathways. This analysis demonstrates that targeting the epigenetic genes in these pathways might be effective for LUAD.

### Understanding the DNA methylated stage-specific networks

Effective drug target identification in a disease now requires incorporating knowledge of the epigenetic genes with knowledge of other biological features. Biological networks help understand and elucidate the roles of the molecular entities individually and collectively. Therefore, the epigenetically modified genes identified in our study were further analyzed in terms of their interaction partners across different stages of LUAD. This network analysis can help to recognize patterns that were not visible by exploring the expression data alone and help to illustrate the conserved and unique patterns across the stages of LUAD. These patterns could then be further validated in laboratories for their efficacy as drug targets.

The missing links and novel genes of Table 6 were identified in subsequent or other LUAD stages. These novel genes were analyzed for their interacting partners. Table 7 shows the common Significant DNA methylated genes across all stages, having interactions with these novel genes (missing links), were: *AJAP1*, *FOXG1*, *GRIK3*, HAND2, *HOXD4*, *PHOX2A* and *PRKC.* Most of these DNA methylated genes were analyzed for their significance in the previous sections. Analyzing the associations of the novel genes identified the following TFs: *c-Jun*, *SMAD1*, *STAT3*, and others genes like *EGFR*, *BCR*, *SUMO1*, *CALM1*, *CUL5, CTNNB1* etc. The TFs identified as novel for a given stage play an important role in cancers [100]. *C-Jun* was identified as important TF in cancer and its subnetwork has been identified in Stages II and III; which was discussed in the previous section. *EGFR* mutations were associated with NSCLC (http://www.egfr.org). *CALM1* and *CTNNB1* were studied in NSCLC and lung cancer [101,102]. This brief analysis elucidates that novel genes interacting with epigenetic genes can play an important role in LUAD; further highlighting that it is essential to understand stage-specific networks.

To understand the commonality and uniqueness of the Significant DNA methylated genes in the context of the other significant expressed genes, we developed and performed subnetwork analysis (as described in the methodology section). Subnetworks of each size were analyzed with respect to their hub genes. Table 8 shows the hub gene profile across different stages in the size four subnetworks. From this table, it can be seen that *UBC* and *CUL1* were hub genes across top ranked stage-specific subnetwork; *COPB1*, *FOXH1*, *SMAD3, TLR4* for Stages I; *HDAC1* for Stage II. Also *SIRT7*, *SUMO2*, *LY96*, *c-Jun* were hub genes in different stages. Additionally each subnetwork had at least one TF. This analysis also confirms that epigenetic genes are not usually hub genes, but have a direct correlation with TF. Also TFs are usually the hub nodes and play an important role in cancers [100], meaning that targeting DNA methylated genes is advantageous as it would not disrupt the whole network but can induce the necessary changes to restore the functionality.

Analysis of the genes in the subnetworks (Additional file 4) found FOXG1 and PHOX2A (Table 2) to be common across all pathway classes. FOXG1 is already a signature gene for lung cancer [68]. All these subnetworks consisted of at least one TF and these were: *HAND2*, *MYC*, *SMAD2*, *SMAD3*, *SMAD4*, and *TP53;* which are important in cancer [70,100,103-105]. The other genes *AR*, *ATF2*, *CUL1*, *EP300*, *GATA4*, *LEF1*, *SKP2* in these subnetworks have also been identified as important in lung cancer [106-112]. Our analysis identified the highly conserved common subnetworks of GRIK2, *GRIK3*, *GRIK5*, and *GRID2* in the metabolic pathway class. *GRIK3* has been reported to be associated with breast cancer and is also in consideration for diagnostic value in lung cancer [56]. In addition, the analysis also identified some subnetworks with novel genes (see Table 6). This analysis suggests epigenetic genes can be used to target lung cancer genes and identification of epigenetic subnetworks can aid in stage-wise characterization of LUAD.

The top ranked subnetworks of size four in each stage were propagated based on their *SubnetworkStrength* to identify the largest conserved subnetwork across stages. Analysis of the different subnetworks found that a set of seven genes was conserved across the stages. The size of the subnetworks with these conserved genes was 11, and the seven genes were: *UBC*, *KRAS*, *PIK3CA*, *PIK3R3*, *RAF1*, *BRAF*, and *RAP1A*. From Table 8, it was seen that *UBC* was the hub gene in all stages. *UBC* is considered to be a reference gene for lung cancer, though it interacts with important cancer genes like *EGFR*, *PCNA*, *IRAK1*, and *P53*[113,114]. Since *UBC* is involved in ubiquitination, it is responsible for cell death and general maintenance. In this analysis, *UBC* expression was found in all stages, suggesting that its function if disrupted can result in uncontrolled cell division, a key feature of cancer. The *BRAF* gene encodes a *RAS* regulated kinase that mediates cell growth. Recent studies have identified BRAF mutations in NSCLC [115]. Phosphatidylinositol 3′-kinase (*PI3K*) is a heterodimer that consists of catalytic and regulatory subunits. *PIK3CA* is one of the catalytic subunit genes and *PIK3R3* is one of the regulatory subunit genes: both of these genes were present in the conserved subnetwork. *PIK3CA* mutations have been identified in many cancers. The *PIK3CA* pathway consist of the *KRAS* and *EGFR* genes which are important targets for many cancers [100], mutations of *PIK3CA* have been also identified in lung cancer [116]. *PIK3R3* expressions have been associated with cancers like glioblastoma and ovarian cancer in prior studies [117], and recent studies have identified *PIK3R3/AKT* as the target of lung cancer molecule *miR-7* which affects *TLR9* signaling (*TLR* was discussed in a previous section) [118]. This analysis of the conserved seven genes in the DNA methylated genes subnetworks of size 11 elucidated that methylation can affect important LUAD genes and enrichment analysis described the important biological processes associated with these subnetworks. From Table 9, it can be seen that these subnetworks affect the important signaling and metabolic cancer processes. Additional file 4 list the DNA methylated genes associated with these subnetworks. Therefore, it can be concluded that further laboratory validation of epigenetic genes in these conserved subnetworks might be useful in recognizing a novel target of LUAD that can be universal to all stages.

Effectiveness of methodology to extract significant subnetworks for networks obtained using variable dataset sources is proved by the comparison results showed in Table 10 and Figure 6. In addition, the same set of conserved genes was identified by the algorithm which proves the robustness of the analysis pipeline.

## Conclusions

The study was entirely based on the available TCGA data, which has the limitation of unequal samples; still we were able to prove the advantage of integrating epigenetic data, expression data and protein-protein interaction knowledge for advancing of systematic understanding of LUAD. This understanding can be further improved by incorporating the system biology approach to the epigenetic profile across the different stages of LUAD. The study identified 72, 93 and 170 epigenetic genes across Stages I, II and III. A set of 34 common epigenetic genes were identified across the three stages, and it was observed that methylation patterns were similar across Stages I and III, but were different in Stage II. The study also identified known, and novel epigenetic genes across stages that were important in LUAD, these genes could be further validated in the laboratory for their scope as targets. The novel epigenetic genes identified were *PTGDR*, *POU4F2*, *TLX3*, and *MMP26* along with the study identified early and late expression profiles of *NEUROG1*, *AJAP1*, *and CORO6* in LUAD. System biology approach stated that epigenetic genes were not the hub nodes but could still affect the hub genes in the networks, eventually playing a critical role in the disease mechanism. Subnetworks of size 11 with seven conserved genes across the three stages were literature validated, confirming their importance in LUAD. Therefore, it can be concluded that integrating methylated data with expression data can be useful for comprehending in-depth disease mechanism and for the ultimate goal of better target identification.

## Methods

The gene expression and DNA methylation data for LUAD were downloaded from TCGA [119]. The gene expression data were generated by UNC AgilentG4502A_07_3, and the methylation profiles were generated by Illumina HumanMethylation27 DNA Analysis which contains 27,578 CpG dinucleotides in 14,495 genes. These datasets were downloaded on 10-12-2012 and segregated with respect to the stages in LUAD. The protein-protein interaction was downloaded from BioGRID [34]. The dataset from BioGRID comprised of 15,550 proteins and 86,344 interactions. In addition to protein-protein interactions, manually curated human signaling network [120-123] was used to verify the effectiveness of the analysis pipeline. The signaling network consisted of ~6,300 proteins and 63,000 signaling relations (http://www.bri.nrc.ca/wang/).

**Figure 7:**
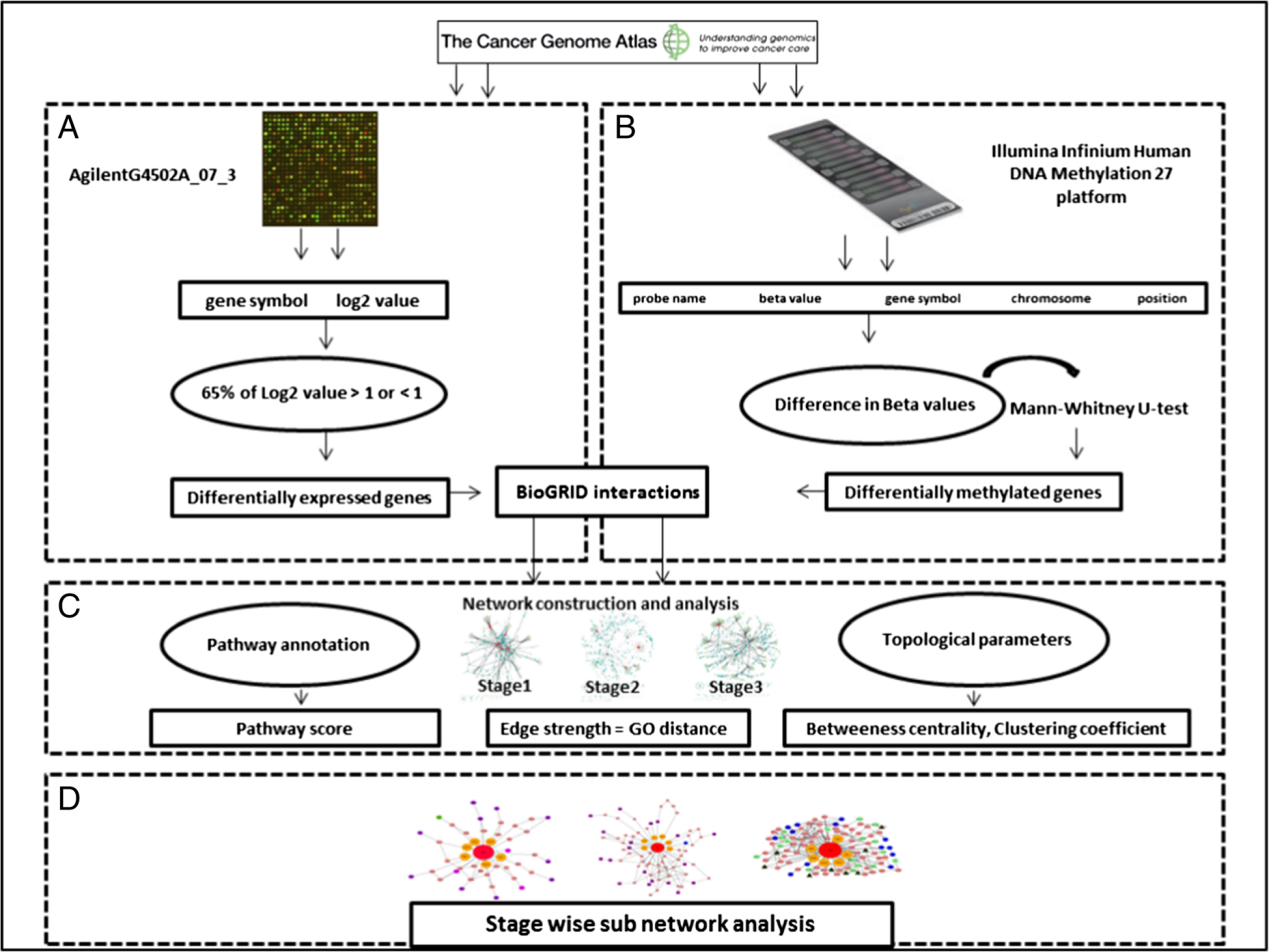
**Overall methodology. (A)** UNC AgilentG4502A_07_03 gene expressed data was analyzed based on the log_2_ values to obtain the differentially expressed genes. **(B)** The methylation data from Illumina HumanMethylation27 were classified for each stage. Significantly DNA methylated genes were identified. **(C)** Stage-specific interaction networks were constructed and annotated with their DNA methylated genes. The nodes and edges of each network were scored based on the topological and biological features. **(D)** The epigenetic subnetworks were identified and compared across stages to understand the epigenetics commonality and uniqueness.

The overall methodology for the stage-wise identification of LUAD process is shown in Figure 7, and it includes four steps (A-D) as given below;

Step A: the gene expression data from UNC AgilentG4502A_07_3 were analyzed based on the log_2_ values to obtain the differentially expressed genes.

Step B: the methylation data from Illumina HumanMethylation27 for each stage were analyzed based on the beta value to obtain the differentially methylated genes.

Step C: the data obtained from Step A and B was integrated to obtain a stage-specific network of LUAD. This network was annotated with the topological and biological features for analyzing the methylated patterns.

Step D: the stage-specific subnetworks were obtained for LUAD.

Following sections contain details of each step.

### Step A. Identification of significant genes from expression data

The level 3 data available from TCGA [119] was segregated based on the stages provided in Metadata. If a gene was *log*_2_ ≥ 1.4 *or* ≤ - 1.4, then the gene was considered for further analysis as it obeyed the stringency with respect to fold change > 2.5 (a log_2_ ratio of 1 represents a 2-fold change) [124]. These genes were termed as “Significant expressed genes”. The average value for each of these genes was then computed and considered for the next level analysis. If a gene was represented by two or more probes, then the median of its expression value was used.

### Step B.1. Identification of significant DNA methylated genes from methylated data

The beta-values [125], for normal and disease samples were downloaded from the TCGA for Illumnia HumanMethylation27 and stratified across stages. The difference between the normal and the disease beta-values were then calculated. Genes with beta-values greater than 0.25 and those with beta-values less than -0.25 were considered for further analysis and were classified as hyper and hypomethylated [125]. For the study, the analysis of *q-value* and 1% FDR gave threshold for the *p-values* obtained in respective stages [32]. The threshold was then used to filter the data and significant DNA methylated genes were derived using the Mann–Whitney U test [126], *p-values* were computed for each gene. Mann–Whitney U test was considered as it can handle variance for unequal sample sizes [127]. These genes were termed as “Significant DNA methylated genes”. Since the sample sizes were small, to get true inferences resampling technique was performed. The samples were permuted large number of times (1000), and Mann Whitney Test was performed on them [128] to get *p-values*[119].

### Step B.2. Analysis of DNA methylated genes with respect to the CpG islands, promoter regions, transcription factors, chromosome distribution and pathways

The correlation of DNA methylated genes with CpG islands is assessed by mapping the position of the DNA methylated gene to CpG position using computational methods [17]. For this study, the significant DNA methylated genes from each stage in LUAD as identified in the previous steps, were mapped to the CpG islands provided by Illumnia HumanMethylation27. These were then classified as TRUE or FALSE based on their location inside or outside of the CpG islands. The CpG islands were then correlated to the promoter region by computing the distance between the transcriptional start sites (TSS) (http://genome.ucsc.edu) and the promoter region. For this study, the promoter region was defined as -1500 to +500 bp around the TSS site [18]. The Significant DNA methylated genes were also analyzed for their transcription functions using Gene Ontology [121] and chromosome distribution (http://www.genome.ucsc.edu). This analysis gave the profile of Significant DNA methylated genes as TFs and their chromosome distribution. To understand the stage-wise profile of pathways consisting of Significant DNA methylated genes, these were then annotated with respect to their pathway association using KEGG [74,129]. This analysis found common and unique pathways across stages.

### Step C. Understanding the stage-specific networks of LUAD

To understand the significance of the DNA methylated genes in LUAD, stage-specific networks were obtained using the following steps:

### Identification of gene-gene interactions and DNA methylated-gene interactions from BioGRID and constructing the stage-specific networks of LUAD

The gene-gene physical interactions (associations) for all the Significant expressed genes and Significant DNA methylated genes were identified using BioGRID for all stages [34]. The networks for each stage were constructed based on these interactions. The nodes of the network were genes and the interactions between them were the edges. The nodes and edges were then analyzed to capture the commonality and differences across the stages. These were computed based on the following criteria: (i) Identifying edges (interaction) between two Significant DNA methylated genes (nodes); (ii) Identifying edges (interaction) between Significant DNA methylated gene (node) and Significant expressed gene (node); (iii) Identifying edges (interaction) between the Significant DNA methylated gene (node) and another gene (node) other than the significant expressed and DNA methylated genes in the given stage. This interaction was termed as the “missing link” and the gene as “novel gene”. The expression pattern of this novel gene was then evaluated in the previous or subsequent stages. The significance of novel genes with respect to LUAD was validated using Biomedical literature.

To understand the overall profile of each stage-specific network of LUAD, a system’s biology approach was developed. All the nodes (genes) and edges (interactions) were annotated with their respective topological and biological features. The statistical computing tool R (http://www.r-project.org) was used to compute the topological features of *betweeness* and *clustering coefficient.* The two biological features considered for the analysis were: *Pathway Significance Score* and *Gene Ontology Semantic Similarity Score*. The *Pathway Significance Score* was based on the occurrence of the given gene in a pathway class. For the study, the KEGG pathways were classified in the three pathway classes and these were (i) the lung cancer pathways, (ii) other cancer pathways (not lung cancer), (iii) other pathways [129]. Each node (gene) in the network was annotated with *betweeness*, *clustering coefficient* and *Pathway Significance Score*. These features were normalized individually and the average of these features was computed. This average was termed as *NodeStrenght*, given as:

(i) $$\begin{array}{l} \mathrm{NodeStrengt}\\ \;h_{v}=\frac{\left(\mathrm{Betweenness}+\mathrm{Clustering}\;\mathrm{coefficient}+\mathrm{Pathway}\;\mathrm{Significance}\;\mathrm{Score}\right)}{3}. \end{array}$$

*Betweenness* of a gene *v* was defined as the inverse of the ratio of the total number of shortest paths from gene *s* to gene *t* given by *σ*_*st*_ to the number of total paths passing through gene *v* (*σ*_*st*_ (*v*)) [130]. This was computed as:

(ii) $$\mathrm{Betweenness}\;\left(B_{\mathrm{bet}\;}\left(v\right)\right)=\sum_{s\neq v\neq t}\frac{\sigma_{\mathrm{st}}\;\left(v\right)}{\sigma_{\mathrm{st}}}.$$

*Clustering coefficient (C*_*v*_*)* was defined as a function based on the triplets of the genes in the network, where a triplet consisted of the three genes (nodes) connected by either two open or three closed undirected ties [131]. The clustering coefficient for the genes in the undirected graph (stage- specific network) was computed as:

For a graph *G=(V,E)* consisting of vertices *V* and a set of edges *E*, where *e*_*i,j*_ connects vertex *v*_*i*_ with vertex *v*_*j*_ and the neighborhood *N*_*i*_ for this vertex *v*_*i*_ was defined as:

(iii) $$N_{i}=\left\{v_{j}:e_{\mathrm{ij}}\in E\right\}.$$

And where *k*_*i*_ represents the number of vertices in the neighborhood of *N*_*i*_. The clustering coefficient for this local graph was then computed as:

(iv) $$\mathrm{Clustering}\;\mathrm{coeficeint}\;\left(C_{v}\right)=\frac{2\;|\left\{e_{\mathrm{jk}}:v_{j},v_{k}\in N_{i},e_{\mathrm{jk}}\in E\right\}|}{k_{i}\;\left(k_{i}-1\right)}.$$

#### Pathway sifnificance score

The pathways associated with each nodes *v* (genes) were identified using KEGG [74,129], and *Pathway Sifnificance Score* was computed as;

(v) $$\begin{array}{l} \text{Pathway}\;\text{Significance}\;\text{Scor}e_{v}=\{\mathrm{lo}g_{10}[\left(\frac{\text{frequency}\;\text{of}\;\text{term}}{\text{Total}\;\text{frequency}}\right)*100\\ \;*{\left(\text{Total}\;\text{frequency}\right)}^{\text{strength}}]\}\text{strength}. \end{array}$$

Where, *Pathway Sifnificance Score* determined the level of importance of a gene in the lung cancer pathways, other cancer pathways (not lung cancer pathways) and other pathways (i.e. pathways that are not termed as lung cancer pathways or non-lung cancer pathways) as given by KEGG pathways; *frequency of terms* equaled the count of the gene in lung cancer pathways, other cancer pathways and other pathways; *Total frequency* was equal to the count of the lung cancer pathways, other cancer pathways and other pathways; *Strength* represents the rank of the pathway class to which the gene belongs to in the stage-specific network. For all the stage-specific network lung cancer pathway was given a rank of 3, other cancer pathways were given a rank of 2 and other pathways were given a rank of 1, of which 3 being the rank of the highest importance followed by 2 and 1 being the lowest rank.

#### EdgeStrength

For any two interacting nodes (genes) in the network, *EdgeStrength*was computed based on their *Gene Ontology Semantic Similarity Score.* This was calculated using the GOSemSim package R [132].

All the genes and their edges in the stage-specific network were then annotated with their *NodeStrength* and *EdgeStrength.* The Significant DNA methylated genes were ranked based on their *NodeStrength*. The highly ranked DNA methylated genes were used to identify subnetworks as described in the following section.

### Step D. Identification and scoring of epigenetically relevant subnetworks across stages

To compare and elucidate the interaction network of Significant DNA methylated genes across stages is a hard problem. Therefore, the networks were analyzed using graph techniques by identifying the relevant subnetworks [133-135]. In this work, subnetworks of different sizes were identified and analyzed across the stages to understand the functional importance of the Significant DNA methylated genes. For the study, we define a subnetwork as a group of connected nodes (genes) with at least one Significant DNA methylated gene, where any two associated genes had the *Gene Ontology Semantic Score ≥ 60%*. These were open subnetworks i.e. no size and shape limitation; therefore a large number of subnetworks were identified making it an NP-hard problem. Starting with the top ranked Significant DNA methylated gene as a seed, its associations were identified, propagated based on *Ontology Semantic Similarity Score ≥60%*, and analyzed with respect to the KEGG pathways. All the genes in a given subnetwork were understood based on the four categories: (i) genes identified in cancer pathways other than lung cancer pathways, (ii) genes identified in lung cancer pathways, (iii) genes identified in signaling pathways (not present in (i) and (ii)) and (iv) genes in the metabolic pathways and other pathways. These subnetworks correlate to distinct functions that specify the distinct mechanism that were compared across the stages.

The Significant DNA methylated genes in each stage were ranked based on their beta-value. The Significant DNA methylated gene with the highest beta-value was considered as a SEED. The SEED and expand algorithm was then used to identify the next connecting gene (node) and interaction (edge) based on the *NodeStrength* and *EdgeStrength*. The gene (node) with highest *NodeStrength* was considered as the next gene (node) if it satisfied the *Gene Ontology Semantic Score ≥ 60%* for the *EdgeStrength*. Thus, subnetworks of different sizes were identified and connected in each of the stage-specific network and ranked based on their *SubnetworkStrength* which was computed as;

(vi) $$\begin{array}{l} \mathrm{SubnetworkStrength}\\ \;=\frac{\sum_{i=1}^{i=k}\left(\mathrm{NodeStrength}\right)+\sum_{j=1}^{j=k-1}\mathrm{EdgeStrength}}{\mathrm{Number}\;\mathrm{of}\;\mathrm{Genes}}. \end{array}$$

Where, *i* are genes (nodes), *j* are interactions (edges), and *k* is the number of genes (nodes).

The subnetworks were compared for their commonality and uniqueness across stages to identify those Significant DNA methylated genes that could be potential targets. These were then validated using literature for their importance in LUAD. In order to prove the universal nature of above detailed network analysis, the methodology was repeated for the interacting genes obtained from manually curated Human Signaling Network dataset (http://www.bri.nrc.ca/wang/).

## Abbreviations

TFs: Transcription factors; NSCLC: Non-small cell lung cancer; SCLC: Small cell lung cancer; LUAD: Lung adenocarcinoma.

## Competing interests

The authors declare that they have no competing interests.

## Authors’ contributions

MPP: conceptualizing and developing methodology, analysis of all the algorithm result, writing manuscript. AAD: data collection and analysis, scripting, figures and input for writing. MJP: PI of the project, conceptualizing the objective, writing manuscript, valuable inputs at all the time. All authors read and approved the final manuscript.

## Authors’ information

Akshay Desai and Meeta Pradhan are co-first authors.

## Supplementary Material

Additional file 1 ***p-value *** **profile of original and corrected resampling data for stage I.** Click here for file

Additional file 2 Pathway distribution for DNA methylated gene across stages. Click here for file

Additional file 3 Analysis of subnetworks of size 2, 3, 4, and 5 across stages. Click here for file

Additional file 4 Analysis of common and unique subnetworks of size 4 revealing the significant genes. Click here for file

Additional file 5 Significant DNA methylated genes in the UBC network. Click here for file
