# Supplementary figures and images for: Systems biology approach to stage-wise characterization of epigenetic genes in lung adenocarcinoma
Source: BMC Syst Biol. 2013 Dec 26;7:141. doi: 10.1186/1752-0509-7-141 (PMC3882327; doi:10.1186/1752-0509-7-141)

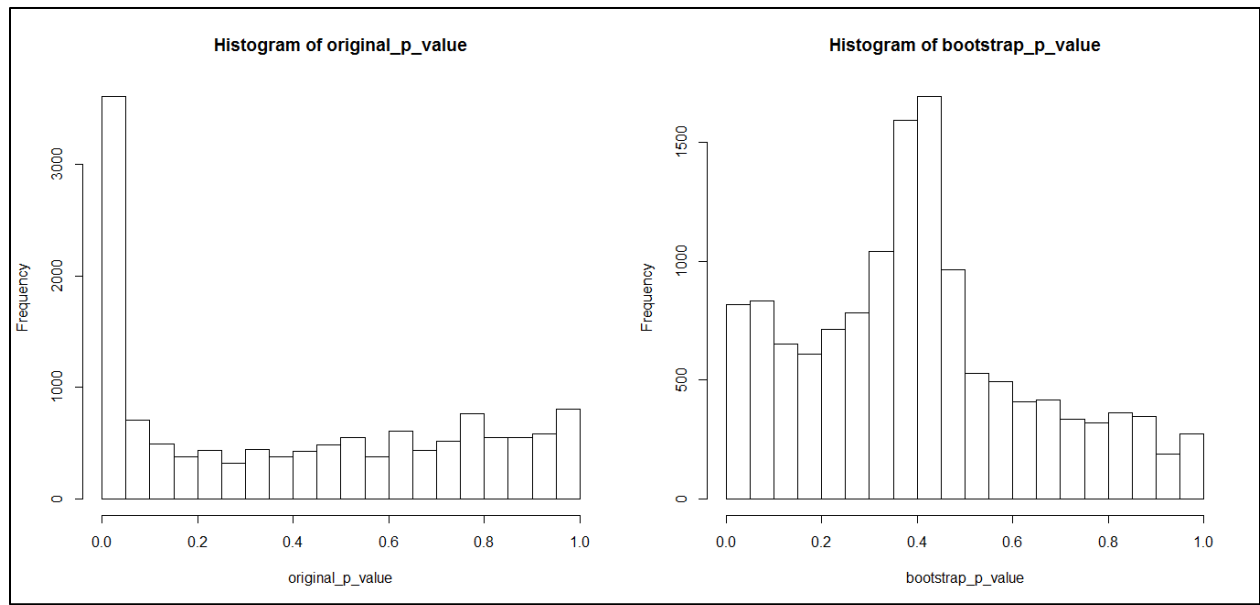

## Appendix I

Supplement: Additional file 1 — p-value profile of original and corrected resampling data for stage I. [file 1752-0509-7-141-S1.pdf]

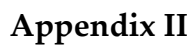

Supplement: Additional file 2 — Pathway distribution for DNA methylated gene across stages. [file 1752-0509-7-141-S2.pdf]
