# Supplementary material for: Systems biology approach to stage-wise characterization of epigenetic genes in lung adenocarcinoma
Source: BMC Syst Biol. 2013 Dec 26;7:141. doi: 10.1186/1752-0509-7-141 (PMC3882327; doi:10.1186/1752-0509-7-141)
Supplement: Additional file 3 — Analysis of subnetworks of size 2, 3, 4, and 5 across stages. [file 1752-0509-7-141-S3.pdf]

Appendix III

Stage	Subnetwork size	Pathway distribution			
		Cancer	Lung cancer	Signaling	Metabolic + others
I	2	1	-	3	5
	3	4	1	5	10
	4	68	45	175	568
	5	2685	1466	5072	12263
II	2	3	1	2	6
	3	58	19	69	107
	4	1176	532	2049	4230
	5	31982	15884	59137	133380
III	2	3	1	3	7
	3	80	33	95	155
	4	1476	679	2578	5952
	5	141149	19951	76205	175691
