# Supplementary material for: Systems biology approach to stage-wise characterization of epigenetic genes in lung adenocarcinoma
Source: BMC Syst Biol. 2013 Dec 26;7:141. doi: 10.1186/1752-0509-7-141 (PMC3882327; doi:10.1186/1752-0509-7-141)
Supplement: Additional file 4 — Analysis of common and unique subnetworks of size 4 revealing the significant genes. [file 1752-0509-7-141-S4.pdf]

Appendix IV: Analysis of common and unique subnetworks of size 4 revealing the significant genes

Pathway	Stages						
	I & II & III	I&II	I&III	II&III	I	II	III
**Cancer**	18	4	43	820	-	336	591
**Lung cancer**	11	-	25	369	-	153	274
**Signaling**	70	2	222	1372	27	641	1049
**Metabolic + others**	74	-	792	2844	135	1347	2677
