# Supplementary material for: Systems biology approach to stage-wise characterization of epigenetic genes in lung adenocarcinoma
Source: BMC Syst Biol. 2013 Dec 26;7:141. doi: 10.1186/1752-0509-7-141 (PMC3882327; doi:10.1186/1752-0509-7-141)
Supplement: Additional file 5 — Significant DNA methylated genes in the UBC network. [file 1752-0509-7-141-S5.pdf]

Appendix V: DNA methylated genes in UBC subnetworks across stages

Stage	UBC interaction with DNA methylated genes
I	*FOXG1, GAS7, HLA-G, HOXD8, LY96, MSC, NPY, PHOX2A*
II	*ACTN2, CDO1, FOXG1, HOXA1, HOXD4, HTR2C, INPP5B, LHX1, NEFH, OTX2, PHOX2A, PRKCB, SERPINB5, SLC6A2, SRGN,* and *TAL1*
III	*ATP6VOD2, CFTR, CRMP1, DGKI, EPO, FLG, HAND2, HOXA7, HOXB4, HOXD4, HOXD12, IHH, INPP5B, NECAB2, NEUROG1, NPY, PDZRN3, POU3F1, PHOX2A, SGMS2, SLC6A2, SPTA1, TBX5, TMEM132D, WNK2* and *XDH*
